# Childlessness, Social Network Profiles in Midlife and Late Adulthood, and Their Implications for Subjective Well-Being

**DOI:** 10.1093/geronb/gbae055

**Published:** 2024-04-03

**Authors:** Bussarawan Teerawichitchainan, Dahye Kim, Christine Ho

**Affiliations:** Department of Sociology and Anthropology, National University of Singapore, Singapore, Singapore; Centre for Family and Population Research, National University of Singapore, Singapore, Singapore; Centre for Family and Population Research, National University of Singapore, Singapore, Singapore; School of Economics, Singapore Management University, Singapore, Singapore; (Social Sciences Section)

**Keywords:** Latent class analysis, Network typology, Singapore

## Abstract

**Objectives:**

Despite the rising prevalence of individuals reaching advanced age without children, little is known about the diversity of support networks *within* childless populations. We examine the network profiles of childless adults aged 50+ in Singapore, which observes high childlessness rates despite societal emphasis on familism.

**Methods:**

We employ latent class analysis to derive network typology based on a 2022 nationwide survey in Singapore. Additionally, we use logistic regression analyses to investigate the sociodemographic correlates of childless individuals’ network types and the associations between these network types and subjective well-being.

**Results:**

Childless Singaporeans form a heterogeneous group characterized by different support networks. Evidence suggests the centrality of parents in the childless’ social networks and the continuity of parent–child support exchanges that extend into the child’s midlife and late adulthood. When parents are absent, siblings/extended kin serve as their support sources. Age, sibship size, and socioeconomic status are key correlates of network types. Membership in diverse networks is beneficial to the subjective well-being of childless individuals. Although one-fifth of childless individuals in restricted networks demonstrate significantly poorer well-being, the remaining four-fifths show comparable, if not better, well-being than the non-childless.

**Discussion:**

Results underscore the importance of differentiating network types among the childless, particularly when assessing their well-being. Contrary to the notion of associating later-life childlessness with social isolation and vulnerabilities, many childless Singaporeans manage to construct non-child-based networks equipped with various supportive relations that cater to their needs. Nevertheless, persistent vulnerabilities among restricted network members deserve policymakers’ attention.

The globally rising trends of childless middle-aged and older adults are historically unprecedented (Verdery et al., 2019). Concerns have been raised regarding their well-being, especially in countries where adult children remain a lynchpin of later-life support (Albertini & Mencarini, 2014; Kreager & Schröder-Butterfill, 2004). Compared to the non-childless, childless older individuals tend to face greater risks of social isolation (Koropeckyj-Cox, 1998), informal care deficits (Deindl & Brandt, 2017), worse physical and psychological health (Feng, 2018; Quashie & Pothisiri, 2018), and institutionalization and premature death (Hsieh & Zhang, 2021). The childless’ vulnerabilities have been linked to their less-endowed social networks (Schnettler & Wohler, 2016; Wenger et al., 2007). Their support networks are typically smaller than those of the non-childless featuring a higher proportion of nonkin and age peers who are deemed less reliable for critical support during declining health (Dykstra, 2006; Vicente & Guadalupe, 2022).

Contributing to this growing literature, our study examines social network profiles of childless Singaporeans aged 50+ based on a recent nationwide survey. We explore whether a social network typology can be discerned among the childless and how their network types are distinct from, or mirror those observed in other populations. We investigate how membership in different network types is linked to the childless’ sociodemographic characteristics. Furthermore, we examine the association between the childless’ network profiles and subjective well-being.

This study expands the literature in three manners. First, we examine heterogeneity in later-life network types *within* the childless population. A few exceptions notwithstanding (e.g., Wu & Pollard, 1998), most research overlooks the diversity among the childless by comparing the social networks of the childless and non-childless. Given their increasing prevalence and number globally, the childless are not an undifferentiated group (Dykstra & Wagner, 2007; Mynarska et al., 2015). They exhibit diverse characteristics, including those who have never been married, married individuals choosing not to have children, and those facing childlessness due to infertility or outliving their children. These varied circumstances may differentially shape their network profiles in midlife and late adulthood.

Next, we utilize a latent class analysis (LCA) to derive network typology. In past research, characteristics (e.g., composition and functions) of the childless’ social networks were often examined separately (Deindl & Brandt, 2017; Dykstra, 2006). Such analytical strategies may not fully capture the complexity of later-life interpersonal environments. Network typology allows researchers to overcome this shortcoming by incorporating a composite collection of network characteristics to parsimoniously describe both structural components (e.g., size) and functional properties (e.g., support type) of a network (Fiori et al., 2006; Litwin, 2001; Wenger, 1991). Although this approach has been increasingly utilized to examine older persons’ network constellations (Guadalupe & Vicente, 2021), little is known about network types among the childless.

Thirdly, our study provides empirical evidence on the network profiles of childless individuals in a less-explored setting. Existing literature has predominantly been theorized based on evidence from western countries, where childlessness is assumed to be more widespread (Kreyenfeld & Konietzka, 2017). With the current total fertility rate of 0.97, Singapore is expected to be the world’s fifth oldest country by mid-century (Malhotra et al., 2019). Presently, nearly 15% of Singaporeans aged 60+ are childless, a rate surpassing that of several western countries (Ho et al., 2023; Sobotka, 2017). Additionally, one in five Singaporeans in their 50s currently does not have children (Yeung & Hu, 2018). The proportions childless are therefore expected to grow steadily, warranting a deeper understanding regarding the support networks of Singaporeans who age without children.

## Literature Review

A social network is an interconnected web of an individual’s social relations, characterized by structural dimensions (e.g., size and composition) and encompassing various types of support (e.g., material and emotional support). Middle-aged and older adults are commonly part of certain network groupings (Berkman, 1983). According to the social convoy model, individuals hold a central position within their social networks depicted as a supportive convoy that encompass family members, friends, neighbors, and significant others (Antonucci & Akiyama, 1987). In the convoy of older persons, adult children typically play a vital role, contributing positively to their well-being. The hierarchical compensatory theory further argues that individuals seek support from different sources in a systematic sequence, depending on the perceived accessibility and dependability of each source (Dykstra, 2016). Evidence from Europe demonstrates that childless older persons typically address the absence of offspring by cultivating meaningful relationships and relying on support from siblings, collateral kin, friends, and neighbors (Deindl & Brandt, 2017; Dykstra, 2006; Schnettler & Wohler, 2016). In instances where such support is lacking, especially due to severe illnesses, recourse to formal assistance from nonprofit organizations and state welfare systems becomes necessary (Albertini & Mencarini, 2014; Wenger, 2009).

Researchers have employed diverse methods to study later-life support networks (Cornwell & Schafer, 2016). Profile-based methods, including cluster analysis and LCA, have been utilized to typologize social networks based on composite indicators such as network size, support sources, frequency, proximity to members, and social participation (Litwin, 2001; Wenger, 1991). Later-life network profiles are consistently found to cluster around four types (Guadalupe & Vicente, 2021). Diverse networks involve broad support from both kin and nonkin with active social participation. Family-focused networks emphasize strong family ties. Friend-focused networks prioritize interactions with friends/neighbors. Restricted networks have few social connections and limited community engagement. Recent evidence highlights contextual and cultural variations in network types. Litwin and Shiovitz-Ezra (2011a), for instance, observe congregant networks among older Americans, whereas Cheng et al. (2009) identify a network type in Hong Kong characterized by frequent support exchanges with distant kin.

Moreover, previous research shows nuanced relationships between sociodemographic characteristics and later-life network types. Older women typically cultivate diverse or friend-focused networks, whereas older men are commonly associated with restricted or family-centered networks (Cheng et al., 2009; Windsor et al., 2016). Evidence further suggests that older cohorts are typically found in network types that contain proportionally more kin than nonkin (Kim et al., 2016). Variations in network types are also found across SES, ethnicities, and religious groups. For instance, higher SES is commonly associated with diverse and friend-oriented networks (Djundeva et al., 2019; Park et al., 2018). Meanwhile, minority status and no religious affiliation increase the likelihood of restricted network membership (Litwin & Shiovitz-Ezra, 2011b).

Network types tend to correlate with later-life well-being (Fiori et al., 2006). The convoy theory posits that individuals are surrounded by a convoy of social relationships offering social support and social control that may influence their well-being (Antonucci & Akiyama, 1987; Berkman, 1983). The task-specific support theory further argues that different network members have specific roles in support provision (Litwak, 1985). For instance, immediate kin offer instrumental help and a sense of belonging, whereas nonkin enhance social integration. Thus, network types characterized by diverse social ties and support from kin and nonkin tend to have the most positive impact on later-life well-being (Djundeva et al., 2019; Park et al., 2018). Friend-focused networks are found beneficial for the well-being of western populations after diverse and family-focused networks (Litwin & Shiovitz-Ezra, 2011a). Meanwhile, networks comprising distant kin demonstrate a positive association with well-being in Asian settings, underscoring the importance of extended family (Cheng et al., 2009). Research further shows that restricted networks typically have adverse effects on later-life well-being (Fiori et al., 2006; Kim et. al., 2016).

### The Singapore Context

Singapore’s 5.9-million population includes 4.1 million citizens and permanent residents. The population is ethnically and religiously diverse, with three-quarters being of Chinese ethnicity, while the remaining minority primarily consists of Malays and Indians. About 80% of Singaporeans reside in government-subsidized high-rise apartment buildings. Over the past five decades, Singapore has experienced significant fertility decline and socioeconomic transformation. Its ultra-low fertility is closely linked to the postponement and decline in marriage. These trends are influenced by a multitude of factors, including increased educational and labor market opportunities for women, rising opportunity costs of childbearing, persistent norms for women to marry up, and the financial burden of educating children (Jones, 2012). Singapore’s past demographic trends have resulted in accelerated population aging and a significant prevalence of childlessness among today’s middle-aged and older populations.

Various policies have been implemented to address the well-being of older Singaporeans (Malhotra et al., 2019). Aligning with prevalent filial piety norms, Singapore’s welfare system promotes familism by emphasizing the roles of adult children in supporting aging parents. For instance, the government provides additional housing subsidies to incentivize Singaporeans to live with or near their parents. The Maintenance of Parents Act allows older Singaporeans to legally pursue support from their children. Moreover, its long-term care policy, funded by individual contributions, government funding, and insurance scheme, focuses on home-based and community-based care. These cultural and institutional emphases on adult children and family members supporting older persons may influence the childless’ later-life network profiles.

Recent research demonstrates the integral role of family relationships in the social networks of older Singaporeans. For example, Sung and colleagues (2022) identify five network types among adults aged 60+, including diverse, unmarried but diverse, extended family, immediate family, and restricted networks. Lau and colleagues (2019) further demonstrate that older Singaporeans maintain strong social connections with family/relatives, while showing relatively less involvement with friends/neighbors. To our knowledge, no empirical studies have specifically identified network types among childless, middle-aged, and older adults or explored their associations with subjective well-being. This research gap warrants attention, especially in rapidly aging populations with high childlessness rates, such as in Singapore.

### Hypotheses

We anticipate that the network profiles of childless, middle-aged, and older Singaporeans will demonstrate both distinctive features and shared characteristics when compared to broader populations in Asian and western contexts. According to the convoy theory, given the importance of adult children in supporting aging parents and the tendency for childless individuals to take on significant parental caregiving roles (Kim et al., 2024; Pesando, 2019), we expect to identify unique network types featuring broad support exchanges between childless individuals and their parents. Additionally, in line with the hierarchical compensatory perspective, we hypothesize the formation of network types characterized by supportive relationships between childless individuals and their siblings/extended kin, particularly among individuals without living parents. Moreover, despite the normative emphasis on family-focused social relations, the absence of child-rearing responsibilities may afford the childless resources to cultivate nonkin relationships and social participation. Hence, we expect to discern networks characterized by active social participation and support exchanges with friends/neighbors among childless individuals.

Furthermore, we anticipate the childless’ network profiles to vary across sociodemographic characteristics, such as age, gender, and socioeconomic status. Finally, we posit that membership in networks with broad support from both kin and nonkin will be most beneficial for the subjective well-being of the childless, while membership in restricted networks will be the most detrimental.

## Method

### Sample

Data are from a nationwide survey of 1,500 Singaporeans aged 50+, with oversampling of childless individuals. The sample was randomly selected from a nationally representative listing of residential households containing at least one member aged 50+. The listing compiled by Singapore’s Department of Statistics utilized a proportionate stratified design to cover diverse dwelling types and socioeconomic characteristics within the community-dwelling populations aged 50+. Because only 1.8% of older Singaporeans are institutionalized, the impact on the national representativeness of the survey is expected to be minimal (Chan et al., 2018). The survey, conducted in 2022, employed face-to-face interviews carried out in respondent homes, each lasting approximately 1 hr. We restrict the analytic sample to childless respondents (*N* = 500).

### Measures

#### Social network variables

Social networks comprise a range of social connections that provide an individual access to social, emotional, and practical support (Gray, 2009). To capture these multiple support dimensions, we incorporate 14 network indicators, which systematically consider whether the respondent exchanges structural, material, and affectual support with four groups of network members (parents, siblings, extended family, friends/neighbors) and the levels of their social participation. We dichotomize each network variable (yes/no). Structural support refers to coresiding with network members at the time of the survey. Coresidence indicates spatial proximity, which facilitates social contacts between respondents and network members (Silverstein & Bengtson, 1997). Material support captures any money/gift exchanges with network members in the previous year. Affectual support refers to feeling emotionally close to network members. Social participation captures weekly involvement in community-based and religious activities.

While acknowledging the significance of spouse in support networks of partnered childless individuals (Connidis & McMullin, 1992), our decision to exclude spouse as a network indicator is based on several reasons. Nearly 80% of our analytic sample are unpartnered. Past research demonstrates that an inclusion of spouse may obscure the significant variations in social networks among unpartnered individuals in Asian settings where extended kinship is valued (Cheng et al., 2009). Summary statistics and detailed definitions of network indicators are shown in Supplementary Table 1.

#### Subjective well-being

We consider four indicators: loneliness, depression, happiness, and life satisfaction. For loneliness and depression, we dichotomize the 4-point answer scale (1 = felt lonely/depressed sometimes or more often, 0 = rarely or never felt lonely/depressed). For happiness, 1 = always felt happy and 0 = occasionally felt happy or less often. These indicators are dichotomously coded because of their skewed distributions. Preliminary analyses treating them as binary versus ordinal measures reveal largely similar associations with network types. Lastly, life satisfaction indicates how satisfied the respondent presently feels towards his/her life from 1 (very dissatisfied) to 5 (very satisfied).

#### Sociodemographic characteristics

We incorporate variables previously associated with network types and subjective well-being: age (50–64/65+), gender (male/female), ethnicity (Chinese/non-Chinese), religion (Buddhism, non-Buddhism, no religion), nativity status (native-born/foreign-born), current marital status (currently married, never married, widowed/divorced/separated), whether the respondent reported childlessness was involuntary (i.e., pregnancy loss, infertility, child death), and sibship size (zero, one-two, three-four, five+). For socioeconomic status, we include education (primary, secondary, postsecondary), work status (currently working/not working), and monthly income (under S$1,000, S$1,000–S$1,999, S$2,000–S$3,999, S$4,000+). Lastly, we consider functional limitation as an indicator of physical health. Several sociodemographic covariates are dichotomously coded based on their distributions and contextual reasons. Sample description by well-being indicators and sociodemographic characteristics is shown in Supplementary Table 2.

### Analyses

We determine network typologies using LCA, which assumes a probabilistic connection between the latent concept (the childless’ social networks) and manifest indicators (the 14 social network variables). The LCA produces a latent, mutually exclusive categorical variable that characterizes qualitative differences between classes. Our sample size (*N* = 500) is appropriate for LCA (Nylund-Gibson & Choi, 2018) and comparable to past research (Fiori et al., 2008; Webster et al., 2015). Once the best-performing LCA model is identified (5-class model in our case), we examine each latent class by its network indicators and assign a group name. Next, we examine bivariate associations between sociodemographic characteristics and the network type variable. Moreover, we create five dichotomous outcome variables for each network type and regress each network outcome on sociodemographic variables using binary logistic regressions. Lastly, we utilize binary and ordered logistic regressions to investigate how network types are associated with subjective well-being net of sociodemographic characteristics—first by comparing well-being differences within the childless population and subsequently by adding the non-childless as the comparison group.

## Results

### Network Profiles of Childless Middle-Aged and Older Adults

The LCA identified the 5-class model as the optimal model based on not only model-fit criteria but also interpretability and parsimoniousness. The model has low Bayesian information criterion (BIC) value, indicating a better fit of the model to the data. Significant results from the Lo-Mendell-Ruben adjusted likelihood ratio test (LMR-LRT) and bootstrapped likelihood ratio test (BLRT) indicate that the *k-class* model fits the data better than the *k −* 1 *class* model. The entropy value of nearly 0.80 meets a conventional cutoff for precise classification quality (see Supplementary Table 3).

Table 1 presents prevalence and distribution of network indicators across childless respondents in the five latent classes. The five network types are labeled as follows: (a) diverse, parental presence, (b) diverse, parental absence, (c) parent-centered, (d) siblings/extended family, and (e) restricted. The first two clusters demonstrate well-endowed networks characterized by diverse kin and nonkin support and higher levels of social participation compared to other network types. The *diverse, parental presence* network type accounts for 18% of the childless and demonstrates relatively high probabilities of coresiding with parents, exchanging material and affectual support with kin and nonkin, and participating regularly in community and religious activities. The *diverse, parental absence* network type, comprising 13% of the sample, shows high probabilities of support exchanges with all network members except for parents because most have no surviving parents. Compared to the former, the latter demonstrates higher probabilities of material and affectual support with kin and nonkin, and religious participation. Nevertheless, they are unlikely to coreside with any network members except for a low probability of living with siblings.

**Table 1.** Distribution and Probabilities of Social Network Indicators Among Childless Individuals (*N* = 500) Across Latent Classes

Social network indicators	Network type				
	Diverse, parental presence	Diverse, parental absence	Parent-centered	Siblings/extended family	Restricted
	(18.0%)	(13.4%)	(16.8%)	(31.8%)	(20.0%)
*Structural support*					
Parents	0.613	0.000	0.500	0.000	0.000
Siblings	0.215	0.158	0.136	0.231	0.000
Extended family	0.070	0.000	0.040	0.095	0.000
Friends/neighbors	0.000	0.000	0.037	0.009	0.069
					
*Material support*					
Parents	0.972	0.059	0.896	0.000	0.037
Siblings	0.672	0.825	0.115	0.336	0.157
Extended family	0.771	0.818	0.161	0.457	0.157
Friends/neighbors	0.369	0.613	0.090	0.072	0.242
					
*Affectual support*					
Parents	0.954	0.007	0.961	0.010	0.000
Siblings	0.723	0.883	0.595	0.765	0.128
Extended family	0.359	0.623	0.308	0.417	0.013
Friends/neighbors	0.453	0.590	0.102	0.184	0.210
					
*Social participation*					
Regular community participation	0.337	0.231	0.114	0.164	0.174
Regular religious participation	0.411	0.621	0.046	0.178	0.191

The *parent-centered* network type, accounting for 17% of childless respondents, is characterized by high probabilities of structural, material, and affectual support exchanges primarily with parents. This class shows low probabilities of exchanging material support with siblings and extended family yet demonstrating moderate probabilities of affectual support with them. Compared to others, this group has the lowest probabilities of social participation and affectual support exchanges with nonkin. The *siblings/extended family* network type is the largest cluster accounting for 32% of the sample. This class has high probabilities of living with and feeling emotionally close to siblings, while experiencing moderate probabilities of material and affectual support exchanges with extended family. Interactions with parents are nearly absent because most have no living parents. This group also demonstrates limited ties with friends/neighbors and comparatively low probabilities of social participation. Finally, the *restricted* network type, accounting for 20% of the sample, is characterized by consistently low probabilities of any support exchanges and social participation. They are likely to have no surviving parents and limited support exchanges with siblings and extended kin. This cluster relies relatively more on nonkin for material and affectual support, although the probabilities of these two support dimensions are lower than those of the *diverse* network types.

### Sociodemographic Correlates of Network Types

Table 2 presents the bivariate associations between sociodemographic characteristics and network types among childless adults. Results show that childless individuals aged 50–64 are more represented in networks characterized by parental presence. Meanwhile, their older counterparts (age 65+) are more prominent in siblings/extended family and restricted networks. Proportionally more men are found in parent-centered and siblings/extended kin networks, whereas women are more represented in the two diverse network types. Likewise, non-Buddhist childless adults prevail in both types of diverse networks. Conversely, childless individuals without religion and those who are foreign-born are more represented in restricted networks.

**Table 2.** Descriptive Statistics, Sociodemographic Characteristics and Network Type Among Childless Individuals (*N* = 500)

Variable categories		Network type					Total	*X* ^2^
		Diverse, parental presence	Diverse, parental absence	Parent-centered	Siblings/extended family	Restricted		
	*N*	%	%	%	%	%	%	
Age								86.14^***^
50–64	311	25.7	12.5	23.8	25.7	12.2	100.0	
65+	189	5.3	14.8	5.3	41.8	32.8	100.0	
Gender								26.28^***^
Male	205	13.7	6.3	22.9	35.6	21.5	100.0	
Female	295	21.0	18.3	12.5	29.2	19.0	100.0	
Ethnicity								0.60
Chinese	416	18.5	13.2	16.6	31.5	20.2	100.0	
Non-Chinese	84	15.5	14.3	17.9	33.3	19.1	100.0	
Nativity status								9.77^**^
Native-born	449	18.7	14.0	17.6	31.2	18.5	100.0	
Foreign-born	51	11.8	7.8	9.8	37.3	33.3	100.0	
Religion								32.52^***^
Buddhism	208	14.9	7.7	21.6	35.6	20.2	100.0	
Non-Buddhism	211	23.2	20.9	12.3	26.5	17.1	100.0	
No religion	81	12.4	8.6	16.1	35.8	27.2	100.0	
Marital status								18.81^**^
Currently married	107	19.6	11.2	26.2	30.8	12.2	100.0	
Never married	336	17.6	15.2	15.2	31.6	20.5	100.0	
Widowed/divorced/separated	57	17.5	7.0	8.8	35.1	31.6	100.0	
Involuntary childlessness								2.15
Involuntary	105	18.1	10.5	14.3	34.3	22.9	100.0	
Other reasons	395	18.0	14.2	17.5	31.1	19.2	100.0	
Sibship size								88.33^***^
Zero	45	6.7	2.2	11.1	15.6	64.4	100.0	
One-two	134	27.6	14.9	20.2	25.4	11.9	100.0	
Three-four	163	19.0	9.2	19.0	33.1	19.6	100.0	
Five or more	158	12.0	19.6	13.3	40.5	14.6	100.0	
Education								84.02^***^
Primary	138	3.6	4.4	18.1	42.0	31.9	100.0	
Secondary	164	15.2	14.6	12.8	40.9	16.5	100.0	
Postsecondary	198	30.3	18.7	19.2	17.2	14.7	100.0	
Work status								28.54^***^
Currently working	288	24.0	14.6	18.1	29.5	13.9	100.0	
Not working	212	9.9	11.8	15.1	34.9	28.3	100.0	
Monthly income								74.66^***^
Under S$1,000	146	6.2	8.2	17.1	38.4	30.1	100.0	
S$1,000–S$1,999	134	12.7	11.2	14.9	36.6	24.6	100.0	
S$2,000–S$3,999	108	22.2	16.7	16.7	32.4	12.0	100.0	
S$4,000+	99	38.4	19.2	18.2	17.2	7.1	100.0	
Functional limitation								6.89
No ADL/IADL limitation	462	18.4	14.1	16.7	32.0	18.8	100.0	
1+ ADL/IADL limitation	38	13.2	5.3	18.4	29.0	34.2	100.0	

*Notes*: ADL = activities of daily living; IADL = instrumental activities of daily living.

^*^ *p* < .10.

^**^ *p* < .05.

^***^ *p* < .01.

Moreover, currently married childless adults are more frequently found in parent-centered networks, whereas unpartnered individuals, especially those who are widowed/divorced, are more present in restricted networks. Similarly, those without siblings are overwhelmingly represented in restricted networks. By contrast, those with large sibship (5+ siblings) are prominent in siblings/extended kin networks. Results further show notable bivariate associations between network types and socioeconomic indicators. The childless with postsecondary education, high income, and being currently employed are found more frequently in diverse network types. Meanwhile, those with low education, low income, and nonwork status are more common in siblings/extended family and restricted networks. Lastly, network types demonstrate no significant bivariate relationships with ethnicity, involuntary childlessness, and physical health status.

Table 3 presents multivariate analyses that regress each network type on sociodemographic variables. Odds ratios indicate the relative likelihood that respondents with a certain sociodemographic characteristic will be situated within a particular network type. Multivariate results are consistent with bivariate results in Table 2. First, age is a key determinant of network types among the childless. Older age (65+ vs 50–64) is significantly associated with a lower likelihood of membership in diverse, parental presence networks and parent-centered networks. Conversely, it is linked to a greater likelihood of having siblings/extended family and restricted networks. Another important determinant is sibship size, which shows a strong negative association with restricted networks, while demonstrating a positive relationship with siblings/extended family networks. Having more siblings partially increases the odds of diverse networks.

**Table 3.** Odds Ratios and Robust Standard Errors From Binary Logistic Regressions Determining Sociodemographic Correlates of Five Network Types

Variable categories	Diverse, parental presence network (*n* = 487)	Diverse, parental absence network (*n* = 487)	Parent-centered network (*n* = 487)	Siblings/extended family network (*n* = 487)	Restricted network (*n* = 487)
	Odds ratio	Odds ratio	Odds ratio	Odds ratio	Odds ratio
Aged 65+ (Ref: 50–64)	0.260^***^ (0.099)	1.574 (0.502)	0.165^***^ (0.064)	2.043^***^ (0.489)	2.227^***^ (0.674)
Female (Ref: Male)	1.522 (0.475)	2.821^***^ (1.033)	0.521^**^ (0.147)	0.784 (0.176)	0.855 (0.238)
Non-Chinese (Ref: Chinese)	0.641 (0.281)	0.565 (0.256)	2.391^*^ (1.082)	1.396 (0.484)	1.037 (0.509)
Foreign-born (Ref: Native-born)	0.392^*^ (0.195)	0.372 (0.264)	0.375^*^ (0.208)	1.877^*^ (0.683)	2.580^**^ (1.069)
Religion (Ref: Buddhism)					
Non-Buddhism	1.547 (0.525)	2.935^***^ (1.043)	0.310^***^ (0.119)	0.719 (0.213)	1.004 (0.374)
No religion	0.413^*^ (0.200)	0.808 (0.425)	0.724 (0.283)	1.427 (0.430)	1.916^*^ (0.720)
Marital status (Ref: Currently married)					
Never married	1.142 (0.427)	0.964 (0.482)	0.397^**^ (0.143)	1.021 (0.328)	1.844 (0.828)
Widowed/divorced/separated	1.713 (0.780)	0.872 (0.625)	0.228^***^ (0.124)	0.983 (0.378)	2.214 (1.261)
Involuntary childlessness (Ref: Other)	1.121 (0.413)	0.805 (0.396)	0.819 (0.311)	1.122 (0.351)	1.043 (0.393)
Sibship size (Ref: Zero)					
One-two	2.994^*^ (1.982)	4.313 (4.695)	1.378 (0.794)	4.430^***^ (2.236)	0.088^***^ (0.041)
Three-four	1.877 (1.253)	3.159 (3.449)	1.372 (0.766)	5.085^***^ (2.465)	0.154^***^ (0.062)
Five or more	1.407 (0.948)	10.019^**^ (10.711)	0.876 (0.495)	5.772^***^ (2.769)	0.082^***^ (0.035)
Education (Ref: Primary)					
Secondary	2.843^**^ (1.482)	2.091 (1.013)	0.823 (0.310)	1.060 (0.284)	0.543^*^ (0.193)
Postsecondary	4.290^***^ (2.285)	2.203 (1.091)	1.776 (0.698)	0.347^***^ (0.110)	0.821 (0.302)
Currently working (Ref: Not working)	1.271 (0.481)	1.159 (0.426)	0.868 (0.282)	0.937 (0.240)	0.867 (0.271)
Monthly income (Ref: Under $1,000)					
$1,000–$1,999	1.631 (0.808)	1.167 (0.526)	0.686 (0.261)	1.178 (0.352)	0.951 (0.337)
$2,000–$3,999	2.297 (1.205)	1.833 (0.928)	0.477^*^ (0.210)	1.129 (0.372)	0.600 (0.254)
$4,000+	4.019^**^ (2.207)	2.444 (1.391)	0.349^**^ (0.177)	0.735 (0.308)	0.357^*^ (0.204)
Functional limitation (Ref: None)	0.818 (0.470)	0.384 (0.300)	1.218 (0.633)	0.605 (0.267)	2.207^*^ (1.022)

*Notes*: Robust standard errors in parentheses.

^*^ *p* < .10.

^**^ *p* < .05.

^***^ *p* < .01.

Socioeconomic indicators are partially, yet consistently, associated with the childless’ network types. Higher socioeconomic status is generally linked to diverse network types. Childless individuals with secondary and postsecondary education demonstrate a greater likelihood of having diverse, parental presence networks compared to those with primary education by 2.8 and 4.3 times, respectively. Higher education is partially associated with decreased odds of siblings/extended family and restricted networks. Meanwhile, individuals with higher incomes are less likely to belong to parent-centered networks. Multivariate results, however, show no significant relationships between work status and network types.

Furthermore, gender, ethnicity, nativity status, religion, and health status are partially associated with network types. Childless women are significantly more likely to have diverse, parental absence networks, yet less likely found in parent-centered networks compared to childless men. Being non-Chinese is linked to a significantly greater chance of parent-centered networks, although no significant associations are found between ethnicity and other network types. Compared to Buddhism, non-Buddhism is significantly associated with a higher likelihood of diverse, parental absence networks and a lower likelihood of parent-centered networks. Having an out-group status, including being foreign-born, having no religion, and having functional limitations, raises the childless’ likelihood of restricted networks.

Marital status and involuntary childlessness demonstrate limited associations with social networks. Only being unpartnered significantly lowers the odds of parent-centered networks. Results show no statistically significant relationships between marital status and other network types. Similarly, there are no significant relationships between involuntary childlessness status and network types.

### Network Types and Subjective Well-being

Tables 4 and 5 present multivariate logistic regressions whereby each well-being indicator is regressed on the network variable. For each outcome, Model 1 considers subjective well-being as a sole function of network type, whereas Model 2 assesses the net association of network type and well-being by controlling for sociodemographic characteristics. Table 4 examines how subjective well-being varies among childless individuals situated in different network constellations, using those in restricted networks as the reference group. Table 5 adds the non-childless as the comparison group to examine whether the childless embedded in a certain network type are more or less vulnerable than the non-childless regarding subjective well-being, and which network memberships among the childless are more prone to well-being advantages/disadvantages relative to the non-childless.

**Table 4.** Odds Ratios and Robust Standard Errors From Binary Logistic Regression and Ordered Logistic Regression Models Determining the Associations Between Network Type and Subjective Well-being Indicators Among Childless Individuals

Variable	Lonely^a^		Depressed^a^		Happy^a^		Life satisfaction^b^	
	Model 1 (*n* = 496)	Model 2 (*n* = 483)	Model 1 (*n* = 497)	Model 2 (*n* = 484)	Model 1 (*n* = 495)	Model 2 (*n* = 482)	Model 1 (*N* = 500)	Model 2 (*n* = 487)
	Odds ratio	Odds ratio	Odds ratio	Odds ratio	Odds ratio	Odds ratio	Odds ratio	Odds ratio
Network type (Ref: Restricted)								
Diverse, parental presence	0.225^***^ (0.093)	0.335^**^ (0.155)	0.220^***^ (0.094)	0.198^***^ (0.098)	2.131^**^ (0.636)	1.545 (0.559)	3.946^***^ (1.229)	2.689^***^ (1.008)
Diverse, parental absence	0.792 (0.273)	1.480 (0.664)	0.345^**^ (0.145)	0.355^**^ (0.176)	4.015^***^ (1.380)	2.967^***^ (1.196)	5.058^***^ (1.697)	3.834^***^ (1.459)
Parent-centered	0.767 (0.249)	1.149 (0.460)	0.413^**^ (0.154)	0.447^*^ (0.194)	1.246 (0.375)	1.018 (0.361)	3.042^***^ (1.036)	2.365^**^ (0.885)
Siblings/extended family	0.899 (0.247)	1.294 (0.427)	0.543^**^ (0.160)	0.590 (0.204)	1.674^**^ (0.435)	1.376 (0.398)	1.989^***^ (0.515)	2.010^**^ (0.592)
Sociodemographic controls^c^	(no)	(yes)	(no)	(yes)	(no)	(yes)	(no)	(yes)

*Notes*: Robust standard errors in parentheses.

^a^Subjective well-being indicators (lonely, depressed, happy) are assessed using binary logistic regressions.

^b^Life satisfaction is assessed using ordered logistic regressions.

^c^Model 1 includes only network type (i.e., zero-order effect). Model 2 incorporates network type and sociodemographic controls, including age, gender, ethnicity, nativity status, religion, marital status, involuntary childlessness status, sibship size, education, work status, income, and functional limitation. Exponentiated coefficients from the sociodemographic controls are presented fully in Supplementary Table 4.

^*^ *p* < .10.

^**^ *p* < .05.

^***^ *p* < .01.

**Table 5.** Odds Ratios and Robust Standard Errors From Binary Logistic Regression and Ordered Logistic Regression Models Determining the Associations Between Network Type and Subjective Well-being Indicators Among Childless and Non-Childless Individuals

Variable	Lonely^a^		Depressed^a^		Happy^a^		Life satisfaction^b^	
	Model 1 (*n* = 1,493)	Model 2 (*n* = 1,444)	Model 1 (*n* = 1,495)	Model 2 (*n* = 1,445)	Model 1 (*n* = 1,493)	Model 2 (*n* = 1,443)	Model 1 (*N* = 1,500)	Model 2 (*n* = 1,450)
	Odds ratio	Odds ratio	Odds ratio	Odds ratio	Odds ratio	Odds ratio	Odds ratio	Odds ratio
Childlessness status and network type (Ref: Non-childless)								
Diverse, parental presence	0.567 (0.205)	0.305^***^ (0.132)	0.610 (0.233)	0.393^*^ (0.204)	1.210 (0.274)	1.688 (0.539)	0.919 (0.208)	1.054 (0.328)
Diverse, parental absence	1.996^**^ (0.567)	1.118 (0.448)	0.959 (0.355)	0.682 (0.351)	2.281^***^ (0.645)	3.071^***^ (1.148)	1.185 (0.307)	1.348 (0.479)
Parent-centered	1.933^**^ (0.502)	1.015 (0.373)	1.148 (0.362)	0.754 (0.343)	0.708 (0.162)	1.140 (0.355)	0.700 (0.194)	1.052 (0.334)
Siblings/extended family	2.267^***^ (0.435)	1.113 (0.372)	1.508^*^ (0.332)	0.995 (0.405)	0.951 (0.163)	1.469 (0.414)	0.452^***^ (0.073)	0.809 (0.223)
Restricted	2.521^***^ (0.579)	1.167 (0.400)	2.776^***^ (0.652)	1.814 (0.762)	0.568^***^ (0.122)	0.990 (0.306)	0.222^***^ (0.052)	0.412^***^ (0.142)
Sociodemographic controls^c^	(no)	(yes)	(no)	(yes)	(no)	(yes)	(no)	(yes)

*Notes*: Robust standard errors in parentheses.

^a^Subjective well-being indicators (Lonely, depressed, happy) are assessed using binary logistic regressions.

^b^Life satisfaction is assessed using ordered logistic regressions.

^c^Model 1 includes only network type (i.e., zero-order effect). Model 2 incorporates network type and sociodemographic controls, including age, gender, ethnicity, nativity status, religion, marital status, involuntary childlessness status, sibship size, education, work status, income, and functional limitation. Exponentiated coefficients from the sociodemographic controls are presented fully in Supplementary Table 5.

^*^ *p* < .10.

^**^ *p* < .05.

^***^ *p* < .01.

Childless individuals in restricted networks are more likely to report loneliness and depression and are less likely to demonstrate happiness and life satisfaction compared to other childless adults (Models 1, Table 4). After introducing sociodemographic controls (Models 2), well-being disadvantages among the restricted networks remain particularly salient for life satisfaction and depression. Disadvantages associated with restricted networks are modest for loneliness and happiness. That is, compared to the restricted networks, only the childless in diverse, parental presence networks are significantly associated with lowered odds of loneliness, while those in diverse, parental absence networks demonstrate a significantly higher likelihood of happiness. Exploratory analyses (not shown) reveal that differences in well-being among childless in “non-restricted” network types (e.g., between the two diverse network types or between parent-centered vs siblings/extended family networks) are less salient.

Compared to the non-childless, childless individuals, particularly members of restricted and siblings/extended family networks, demonstrate a greater likelihood of loneliness and depression, while showing lowered odds of positive well-being (Models 1, Table 5). However, after sociodemographic characteristics are controlled, the childless’ well-being disadvantages relative to the non-childless are no longer evident for loneliness, depression, and happiness. The remaining disadvantage is the reduction in life satisfaction among restricted network members. Furthermore, childless individuals in diverse networks demonstrate a significantly greater likelihood of positive well-being outcomes than the non-childless. Other sociodemographic characteristics being equal, childless individuals in diverse, parental presence networks are less likely to report loneliness and depression, while those in diverse, parental absence networks are more likely to express happiness than the non-childless.

## Discussion

This study utilizes LCA to derive social network profiles of childless, middle-aged, and older Singaporeans. The person-centered approach allows us to discern a robust network typology by simultaneously considering multiple dimensions of support exchanges from different network members and various social participation. Additionally, we examine the sociodemographic correlates of the childless’ network types and their implications for subjective well-being. Evidence demonstrates heterogeneity in network profiles *within* the childless population, which can be characterized as follows: diverse, parental presence; diverse, parental absence; parent-centered; siblings/extended family; restricted.

Distinct from previous research, our results reveal the centrality of parents in network profiles of childless Singaporeans, particularly those below age of 65. Two network types—diverse, parental presence, and parent-centered—are driven by the presence of living parent(s). Members of these networks are comparable in high probabilities of coresidence, material support, and emotional closeness with parents. Yet, they differ regarding the extent of interactions with other network members and social participation. These network profiles expand the convoy model by highlighting the continuity of parent-adult child support exchanges that extend into the child’s late midlife (Antonucci & Akiyama, 1987).

Parents remaining at the core of the childless’ later-life social networks reflect Singapore’s filial piety tradition and policies that position adult children as the frontline of later-life support (Malhotra et al., 2019). Singapore’s housing policy is noteworthy. Unpartnered Singaporeans are eligible to purchase their own government-subsidized apartments only after age 35. Because a significant majority of childless Singaporeans have never been married, these housing regulations coupled with high private-home prices and the common practice of assigning the childless to live with and care for aging parents, may explain the centrality of parents in the network constellations of over one-third of the childless (Kim et al., 2024).

Next, the most common network type, accounting for 32% of the childless, is characterized by moderate ties with siblings and extended kin. Support exchanges with nonkin and social participation are low among members of this network type. In line with the convoy and hierarchical compensatory theories, childless Singaporeans, particularly those without surviving parents, turn to siblings/extended kin to compensate for the absence of their own children. The likelihood of this network type increases for individuals who are older than 65 years and have multiple siblings.

Evidence further suggests that childless Singaporeans maintain social networks in midlife and late adulthood primarily comprised of support exchanges with immediate and extended kin. Family clearly serves as the primary source of support among the childless, although the dimensions, intensity, and specific sources of support may vary across latent classes. We do not identify any network types that are friend-oriented. Although this finding aligns with past research in Singapore (Lau et al., 2019; Sung et al., 2022), it differs from other studies based on western populations, which commonly discern a friend-focused network (Djundeva et al., 2019; Litwin & Shiovitz-Ezra, 2011b). The inclination for interactions with family aligns with Singapore’s norm of filial obligations, where family-focused social relations take precedence over nonkin relationships (Kim et al., 2016; Thang, 2015).

Moreover, one-third of childless respondents have diverse social networks, engaging in active support exchanges with both kin and nonkin, along with frequent social participation. Individuals who are female, have several siblings, and possess high SES are commonly found in these well-endowed network constellations. This finding resonates with recent evidence demonstrating a sizeable proportion of older Singaporeans in diversified networks despite being unmarried (Sung et al., 2022). Nevertheless, we identify one-fifth of childless respondents with restricted networks, characterized by limited support ties with all network members and low social participation. Older age, foreign-born status, lack of religious affiliation, and poor physical health increase the likelihood of restricted networks, while having multiple siblings and higher education significantly lower the risks.

In examining the effects of network types on subjective well-being, our evidence aligns with prior research in western and Asian settings (Cheng et al., 2009; Djundeva et al., 2019), indicating that membership in restricted networks is least beneficial to the well-being of childless individuals, particularly regarding life satisfaction. Supporting the convoy and task-specific support theories, results further suggest that membership in diverse networks is linked to reduced odds of negative well-being and increased odds of positive well-being especially when contrasted with childless individuals in restricted networks.

Furthermore, countering the notion that later-life childlessness is linked to social isolation and vulnerabilities (Chew, 2020), results reveal that the childless with diverse networks fare better than the non-childless with comparable sociodemographic characteristics. These well-being advantages manifest in a decreased likelihood of loneliness and depression and an increased likelihood of happiness. Although one-fifth of childless individuals with restricted networks demonstrate significantly poorer life satisfaction than the non-childless, the remaining four-fifths have comparable, if not better, well-being than their non-childless counterparts. This underscores the importance of differentiating network types among the childless when assessing their well-being.

Although our study improves an understanding about later-life network profiles of the childless in a non-western setting, it has certain limitations. First, due to data unavailability, we can neither differentiate the quantity of material support exchanges nor consider time-based instrumental support (e.g., housework) in LCA. Moreover, considering that many childless respondents who have never been married may not be fully aware of their infertility status, our self-reported measure of involuntary childlessness may be limited in delineating pathways to childlessness and associations with later-life network constellations. Next, although previous research demonstrates that older persons’ social networks may change as they age (Kim et al., 2016; Sung et al., 2022), our cross-sectional data do not allow us to address the dynamic nature of social networks over time. Additionally, we cannot pinpoint causal relationships between network types and subjective well-being as there may exist unobserved characteristics that drive both network membership and well-being.

Despite these limitations, our study provides some policy insights. For example, sibship size is found to improve the childless’ likelihood of diverse social networks. Consistent with the hierarchical compensatory perspective, this evidence underscores the importance of siblings in maintaining healthy later-life social relationships. More policies can therefore be directed to promote not only intergenerational but also intragenerational support exchanges. Nevertheless, relying primarily on siblings to form the childless’ later-life networks may not be sustainable. Singapore’s decades of ultra-low fertility suggest that upcoming cohorts of childless Singaporeans are likely to age with only one or no siblings at all. In the face of frail health, future childless individuals are likely to depend increasingly on extended kin, nonkin, and the state. Policies, therefore, need to heed the implications of childlessness and limited kin availability for later-life support.

In sum, our evidence suggests that childlessness in midlife and late adulthood defines certain opportunities and constraints in social relations (Dykstra, 2006; Vicente & Guadalupe, 2022). Childless Singaporeans form a heterogenous group characterized by different support networks. Results show minimal differences in subjective well-being between the childless and non-childless across various network types. Many childless individuals may effectively adopt compensatory strategies, enabling them to construct non-child-based social networks equipped with supportive relations that cater to their needs. Nevertheless, we identify a sizeable minority of childless adults in restricted networks whose well-being indicators suggest that they are significantly more vulnerable than their childless counterparts in other network types and non-childless individuals.

More attention is thus warranted to further understand the pathways of the childless to restricted networks. Given that older age, lower SES, poor health, and out-group status (e.g., foreign-born) are associated with the likelihood of restricted networks, future research may examine how these factors are compounded to marginalize some childless individuals, limit their social ties, and worsen their well-being. Furthermore, additional research is needed to investigate how the childless’ network constellations change as they age. For example, what may happen to childless individuals in parent-centered networks who sacrifice their social life to care for their parents? After their parents pass away, could they develop strong ties with remaining kin and/or expand their networks to include nonkin? Given the globally increasing prevalence of individuals reaching advanced age without children, mitigating the childless’ pathways to restricted social networks will be an important policy agenda for promoting later-life well-being.

## Supplementary Material

gbae055_suppl_Supplementary_Tables_S1-S5

## References

[CIT0001] Albertini, M., & Mencarini, L. (2014). Childlessness and social networks in later life: New pressures on familistic welfare states? Journal of Family Issues, 35(3), 331–357. 10.1177/0192513X12462537

[CIT0002] Antonucci, T., & Akiyama, H. (1987). Social networks in adult life and a preliminary examination of the convoy model. Journal of Gerontology, 42(5), 519–527. 10.1093/geronj/42.5.519 3624811

[CIT0003] Berkman, L. F. (1983). The assessment of social networks and social support in the elderly. Journal of the American Geriatrics Society, 31, 743–749. 10.1111/j.1532-5415.1983.tb03393.x 6655175

[CIT0004] Chan, A., Malhotra, R., Manap, N., Yi, Y. T., Visaria, A., Cheng, G., Goh, V., Tay, P., Lee, J., & Maulod, A. (2018). Transitions in health, employment, social engagement, and intergenerational transfers in Singapore study (The SIGNS Study)–I: Descriptive statistics and analysis of key aspects of successful ageing. Centre for Ageing Research and Education, Duke-NUS Medical School.

[CIT0005] Cheng, S., Lee, C. K., Chan, A. C., Leung, E. M., & Lee, J. (2009). Social network types and subjective well-being in Chinese older adults. Journals of Gerontology, Series B: Psychological Sciences and Social Sciences, 64B(6), 713–722. 10.1093/geronb/gbp075 19820232

[CIT0006] Chew, S. (2020). Singapore’s elderly orphan: Vulnerable, isolated, and afraid of dying alone. Rice. https://www.ricemedia.co/culture-people-elderly-orphans/

[CIT0007] Connidis, I. A., & McMullin, J. A. (1992). Getting out of the house: The effect of childlessness on social participation and companionship in later life. Canadian Journal on Aging, 11(4), 370–386. 10.1017/s0714980800006899

[CIT0008] Cornwell, B., & Schafer, M. H. (2016). Social networks in later life. In L. K. George & K. F. Ferraro (Eds.). *Handbook of aging and the social sciences* (8th ed., pp. 181–201). Academic Press. 10.1016/B978-0-12-417235-7.00009-3

[CIT0009] Deindl, C., & Brandt, M. (2017). Support networks of childless older people: Informal and formal support in Europe. *Ageing & Society*, 37(8), 1543–1567. 10.1017/s0144686x16000416

[CIT0010] Djundeva, M., Dykstra, P. A., & Fokkema, T. (2019). Is living alone “aging alone?” Solitary living, network types, and well-being. Journals of Gerontology, Series B: Psychological Sciences and Social Sciences, 74(8), 1406–1415. 10.1093/geronb/gby119 30312447 PMC6777768

[CIT0011] Dykstra, P. A. (2006). Off the beaten track: Childlessness and social integration in late life. Research on Aging, 28(6), 749–767. 10.1177/0164027506291745

[CIT0012] Dykstra, P. A. (2016). Aging and social support. In G. Ritzer (Ed.), The Blackwell encyclopedia of sociology (2nd ed., pp. 1–5). Wiley. 10.1002/9781405165518.wbeosa033.pub2

[CIT0013] Dykstra, P. A., & Wagner, M. (2007). Pathways to childlessness and late-life outcomes. Journal of Family Issues, 28(11), 1487–1517. 10.1177/0192513x07303879

[CIT0014] Feng, Z. (2018). Childlessness and vulnerability of older people in China. Age and Ageing, 47, 275–281. 10.1093/ageing/afx137 29096004 PMC6016684

[CIT0016] Fiori, K. L., Antonucci, T. C., & Akiyama, H. (2008). Profiles of social relations among older adults: A cross-sectional approach. Ageing and Society, 28, 203–231. 10.1017/s0144686x07006472

[CIT0015] Fiori, K. L., Antonucci, T. C., & Cortina, K. S. (2006). Social network typologies and mental health among older adults. Journals of Gerontology, Series B: Psychological Sciences and Social Sciences, 61(1), P25–P32. 10.1093/geronb/61.1.p25 16399938

[CIT0017] Gray, A. (2009). The social capital of older people. Ageing & Society, 29(1), 5–31. 10.1017/s0144686x08007617

[CIT0018] Guadalupe, S., & Vicente, H. T. (2021). Social network typologies of older people: A cross-national literature review. Ciencia & Saude Coletiva, 26, 5133–5148. 10.1590/1413-812320212611.3.23072019 34787205

[CIT0019] Ho, C., Teerawichitchainan, B., Tan, J., & Tan, E. R. (2023). Risk attitudes in late adulthood: Do parental status and family size matter? Research on Aging, 45(5–6), 423–427. 10.1177/01640275221116091 35998085

[CIT0020] Hsieh, N., & Zhang, Z. (2021). Childlessness and social support in old age in China. Journal of Cross-Cultural Gerontology, 36, 121–137. 10.1007/s10823-021-09427-x 33683554 PMC8906474

[CIT0021] Jones, G. (2012). Population policy in a prosperous city-state: Dilemmas for Singapore. Population and Development Review, 38(2), 311–336. 10.1111/j.1728-4457.2012.00494.x

[CIT0022] Kim, B., Park, S., & Antonucci, T. (2016). Longitudinal changes in social networks, health and wellbeing among older Koreans. Ageing & Society, 36(9), 1915–1936. 10.1017/S0144686X15000811

[CIT0023] Kim, D., Ho, C., & Teerawichitchainan, B. (2024). Childlessness and sibling positioning in upward intergenerational support: Insights from Singapore. Journal of Marriage and Family, 86, 526–542. 10.1111/jomf.12965

[CIT0024] Koropeckyj-Cox, T. (1998). Loneliness and depression in middle and old age: Are the childless more vulnerable? Journals of Gerontology, Series B: Psychological Sciences and Social Sciences, 53B(6), S303–S312. 10.1093/geronb/53b.6.s303 9826972

[CIT0025] Kreager, P., & Schröder-Butterfill, E. (2004). Ageing without children: European and Asian perspectives on elderly access to support networks. Berghahn Books. 10.2307/j.ctv287sfsb

[CIT0026] Kreyenfeld, M., & Konietzka, D. (Eds.). (2017). Childlessness in Europe: Contexts, causes, and consequences. Routledge. 10.1007/978-3-319-44667-7

[CIT0027] Lau, Y. W., Vaingankar, J. A., Abdin, E., Shafie, S., Jeyagurunathan, A., Zhang, Y., Magadi, H., Ng, L. L., Chong, S. A., & Subramaniam, M. (2019). Social support network typologies and their association with dementia and depression among older adults in Singapore: A cross-sectional analysis. BMJ Open, 9(5), e025303. 10.1136/bmjopen-2018-025303 PMC6549623 31154300

[CIT0028] Litwak, E. (1985). Helping the elderly: The complementary roles of informal networks and formal system. Guilford.

[CIT0029] Litwin, H. (2001). Social network type and morale in old age. Gerontologist, 41(4), 516–524. 10.1093/geront/41.4.516 11490050

[CIT0030] Litwin, H., & Shiovitz-Ezra, S. (2011a). Social network type and subjective well-being in a national sample of older Americans. Gerontologist, 51(3), 379–388. 10.1093/geront/gnq094 21097553 PMC3095651

[CIT0031] Litwin, H., & Shiovitz-Ezra, S. (2011b). The association of background and network type among older Americans: Is “who you are” related to “who you are with?” Research on Aging, 33(6), 735–759. 10.1177/0164027511409441

[CIT0032] Malhotra, R., Bautista, M., Muller, A., Koh, G., Theng, Y., Hoskins, S., Wong, C., Miao, C., Lim, W., Malhotra, C., & Chan, A. (2019). The aging of a young nation: Population aging in Singapore. Gerontologist, 59(3), 410–410. 10.1093/geront/gny160 30517628

[CIT0033] Mynarska, M., Matysiak, A., Rybińska, A., Tocchioni, V., & Vignoli, D. (2015). Diverse paths into childlessness over the life course. Advances in Life Course Research, 25, 35–48. 10.1016/j.alcr.2015.05.003

[CIT0034] Nylund-Gibson, K., & Choi, A. Y. (2018). Ten frequently asked questions about latent class analysis. Translational Issues in Psychological Science, 4(4), 440–461. 10.1037/tps0000176

[CIT0035] Park, N. S., Jang, Y., Lee, B. S., Chiriboga, D. A., Chang, S., & Kim, S. Y. (2018). Associations of a social network typology with physical and mental health risks among older adults in South Korea. Aging & Mental Health, 22(5), 631–638. 10.1080/13607863.2017.1286456 28290722

[CIT0036] Pesando, L. M. (2019). Childlessness and upward intergenerational support: Cross-national evidence from 11 European countries. Ageing and Society, 39(6), 1219–1254. 10.1017/S0144686X17001519 31130759 PMC6532053

[CIT0037] Quashie, N. T., & Pothisiri, W. (2018). Parental status and psychological distress among older Thais. Asian Social Work and Policy Review, 12(3), 130–143. 10.1111/aswp.12145

[CIT0038] Schnettler, S., & Wohler, T. (2016). No children in later life, but more and better friends? Substitution mechanisms in the personal and support networks of parents and the childless in Germany. Ageing & Society, 36(7), 1339–1363. 10.1017/S0144686X15000197

[CIT0051] Silverstein, M., & Bengtson, V. (1997). Intergenerational solidarity and the structure of adult child-parent relationships in American families. *American Journal of Sociology*, 103(2), 429–460.

[CIT0039] Sobotka, T. (2017). Childlessness in Europe: Reconstructing long-term trends among women born in 1900–1972. In M. Kreyenfeld & D. Konietzka (Eds.), Childlessness in Europe: Contexts, causes, and consequences (pp. 17–53). Routledge. 10.1007/978-3-319-44667-7_2

[CIT0041] Sung, P., Malhotra, R., Cheng, G., & Chan, A. (2022). Transitions in social network types over time among older adults. Gerontology, 68(7), 817–828. 10.1159/000521213 35026756

[CIT0042] Thang, L. L (2015). Social networks and the wellbeing of older adults in Singapore. In S. Cheng, I. Chi, H. H. Fung, L. W. Li, & J. Woo (Eds.), Successful aging: Asian perspectives (pp. 147–163). Springer. 10.1007/978-94-017-9331-5_9

[CIT0043] Verdery, A., Margolis, R., Zhou, Z., Chai, X., & Rittirong, J. (2019). Kinlessness around the world. Journals of Gerontology, Series B: Psychological Sciences and Social Sciences, 74(8), 1394–1405. 10.1093/geronb/gby138 30423167 PMC6777763

[CIT0044] Vicente, H. T., & Guadalupe, S. (2022). Childlessness, personal social networks and wellbeing at advanced ages: A cross-sectional study in a southern European familistic welfare state. Ageing & Society, 44, 597–621. 10.1017/s0144686x22000320

[CIT0052] Wenger, G.C. (2009). Childlessness at the end of life: Evidence from rural Wales. *Ageing & Society*, 29(8), 1243–1259. 10.1017/S0144686X09008381

[CIT0045] Webster, N., Antonucci, T., Ajrouch, K., & Abdulrahim, S. (2015). Social networks and health among older adults in Lebanon: The mediating role of support and trust. Journals of Gerontology, Series B: Psychological Sciences and Social Sciences, 70(1), 155–166. 10.1093/geronb/gbu149 25324295 PMC4861646

[CIT0046] Wenger, G. C. (1991). A network typology: From theory to practice. Journal of Aging Studies, 5(2), 147–162. 10.1016/0890-4065(91)90003-b

[CIT0047] Wenger, G. C., Dykstra, P. A., Melkas, T., & Knipscheer, K. (2007). Social embeddedness and late-life parenthood. Journal of Family Issues, 28(11), 1419–1456. 10.1177/0192513X07303895

[CIT0048] Windsor, T. D., Rioseco, P., Fiori, K. L., Curtis, R. G., & Booth, H. (2016). Structural and functional social network attributes moderate the association of self-rated health with mental health in midlife and older adults. International Psychogeriatrics, 28(1), 49–61. 10.1017/S1041610215001143 26205193

[CIT0049] Wu, Z., & Pollard, M. S. (1998). Social support among unmarried childless elderly persons. Journals of Gerontology, Series B: Psychological Sciences and Social Sciences, 53B(6), S324–S335. 10.1093/geronb/53b.6.s324 9826974

[CIT0050] Yeung, J. W. J., & Hu, S. (2018). Continuity and change in Singapore’s population and Families. In J. W. J. Yeung & S. Hu (Eds.), Family and population changes in Singapore: A unique case in the global family change (pp. 1–26). Routledge.

