# Supplementary material for: Childlessness, Social Network Profiles in Midlife and Late Adulthood, and Their Implications for Subjective Well-Being
Source: J Gerontol B Psychol Sci Soc Sci. 2024 Apr 3;79(6):gbae055. doi: 10.1093/geronb/gbae055 (PMC11091480; doi:10.1093/geronb/gbae055)
Supplement: gbae055_suppl_Supplementary_Tables_S1-S5 [file gbae055_suppl_supplementary_tables_s1-s5.docx]

***The Journals of Gerontology, Series B: Psychological Sciences and Social Sciences* Supplementary Material: Teerawichitchainan, B., Kim, D., Ho, C. Childlessness, Social Network Profiles in Midlife and Late Adulthood, and Their Implications for Subjective Well-being.**

| **Supplementary Table 1**. Summary Statistics of Social Network Indicators. | | | |
| --- | --- | --- | --- |
|  | | | |
| Social network indicators | Childless individuals  (N=500) | Non-childless individuals  (N=1000) | Difference |
| Structural support^a^ |  |  |  |
| Parents | 19.8% | 3.7% | * |
| Siblings | 15.8% | 1.6% | * |
| Extended family | 5.0% | 0.5% | * |
| Friends | 2.2% | 0.5% | * |
| Material support^b^ |  |  |  |
| Parents | 34.6% | 25.2% | * |
| Siblings | 39.6% | 26.2% | * |
| Extended family | 46.0% | 39.4% | * |
| Friends | 23.8% | 14.1% | * |
| Affectual support^c^ |  |  |  |
| Parents | 34.2% | 29.1% | * |
| Siblings | 62.2% | 73.3% | * |
| Extended family | 33.8% | 41.0% | * |
| Friends | 28.2% | 29.0% |  |
| Social participation |  |  |  |
| Community participation^d^ | 20.0% | 16.1% |  |
| Religious participation^e^ | 26.4% | 22.5% |  |
| Note: * Mean differences statistically significant at p<0.05. | | | |
| ^a^ Structural support refers to coresiding with respective network members at the time of the survey. | | | |
| ^b^ Material support refers to exchanging any money/gifts during the past 12 months with respective network members. | | | |
| ^c^ Affectual support with parents refers to whether the respondent feels emotionally satisfied with the relationship with any of their living parents. For siblings and extended family, affectual support refers to whether the respondent feels emotionally close to most or all of their siblings/extended family. Affectual support with friends refers to whether the respondent has three or more friends whom they feel at ease talking about private matters with. Affectual support is measured using various questionnaire items because there is no common measure available in the survey that is applicable for all network members. | | | |
| ^d^ Community participation refers to at least weekly participation in community-based activities (e.g., volunteering, helping neighbors, joining social clubs). | | | |
| ^e^ Religious participation refers to at least weekly attendance of religious services. | | | |

| **Supplementary Table 2**. Sample Description, Childless Individuals (N=500). | | | |
| --- | --- | --- | --- |
|  | | | |
| Variables | Categories | *N* | %/Mean (SD) |
| Feeling lonely^a^ | Rarely/None | *363* | 73.2 |
|  | Sometimes/Occasionally/Always | *133* | 26.8 |
| Feeling depressed^a^ | Rarely/None | *405* | 81.5 |
|  | Sometimes /Occasionally/Always | *92* | 18.5 |
| Feeling happy^a^ | Rarely/None/Sometimes/Occasionally | *232* | 46.9 |
|  | Always | *263* | 53.1 |
| Life satisfaction (1-5) | 1 (very dissatisfied), 2 (dissatisfied), 3 (neither satisfied nor dissatisfied), 4 (satisfied), 5 (very satisfied). | *500* | 3.8 (0.7) |
| Age | 50-64 | *311* | 62.2 |
|  | 65+ | *189* | 37.8 |
| Gender | Male | *205* | 41.0 |
|  | Female | *295* | 59.0 |
| Ethnicity | Chinese | *416* | 83.2 |
|  | Non-Chinese | *84* | 16.8 |
| Nativity status | Native-born | *449* | 89.8 |
|  | Foreign-born | *51* | 10.2 |
| Religion | Buddhism | *208* | 41.6 |
|  | Non-Buddhism (Christianity, Islam, Hindu, Other religions) | *211* | 42.2 |
|  | No religion | *81* | 16.2 |
| Marital status | Currently married | *107* | 21.4 |
|  | Never married | *336* | 67.2 |
|  | Widowed/divorced/separated | *57* | 11.4 |
| Involuntary childlessness | Involuntary | *105* | 21.0 |
|  | Other reasons | *395* | 79.0 |
| Sibship size | Zero | *45* | 9.0 |
|  | One-Two | *134* | 26.8 |
|  | Three-Four | *163* | 32.6 |
|  | Five or more | *158* | 31.6 |
| Education | Primary | *138* | 27.6 |
|  | Secondary | *164* | 32.8 |
|  | Post-secondary | *198* | 39.6 |
| Work status | Currently working | *288* | 57.6 |
|  | Not working | *212* | 42.4 |
| Monthly income^a^ | Under S$1,000 | *146* | 30.0 |
|  | S$1,000-S$1,999 | *134* | 27.5 |
|  | S$2,000-S$3,999 | *108* | 22.2 |
|  | S$4,000+ | *99* | 20.3 |
| Functional limitation | No ADL/IADL limitation | *462* | 92.4 |
|  | 1+ ADL/IADL limitation | *38* | 7.6 |
| Note: ADL=Activities of daily living, IADL=Instrumental Activities of Daily Living | | | |
| ^a^ Due to missing data, category totals vary: feeling lonely, *N* = 496; feeling depressed, *N* = 497; feeling happy, *N* = 495; monthly income, *N* = 487 | | | |

| **Supplementary Table 3**. Model Fit Indices for Latent Classes among Childless Individuals (N=500). | | | | | |
| --- | --- | --- | --- | --- | --- |
|  |  |  |  |  |  |
|  | 3 Class | 4 Class | **5 Class^b^** | 6 Class | 7 Class |
| AIC^a^ | 6670.666 | 6613.854 | **6583.994** | 6565.066 | 6550.805 |
| BIC^a^ | 6856.109 | 6862.516 | **6895.875** | 6940.166 | 6989.124 |
| SABIC^a^ | 6716.45 | 6675.246 | **6660.995** | 6657.674 | 6659.022 |
| p(LMR-LRT)^a^ | 0.2758 | 0.6785 | **0.0378** | 0.1257 | 0.1986 |
| p(BLRT)^a^ | <0.0001 | <0.0001 | **<0.0001** | <0.0001 | <0.0001 |
| Entropy | 0.823 | 0.792 | **0.785** | 0.807 | 0.826 |
| Note:  ^a^ AIC, Akaike information criterion. BIC, Bayesian information criterion. SABIC, Sample size adjusted Bayesian information criterion. p(LMR-LRT), p value from Lo-Mendell-Rubin adjusted likelihood ratio test. p(BLRT), p value from Bootstrapped likelihood ratio test. | | | | | |
| ^b^ Holistically taking fit statistics, interpretability, and parsimoniousness into consideration, we select the 5-class model as the best-performing solution. While the 3-class and 4-class models have lower BIC scores and slightly higher entropy, they do not pass the Lo-Mendell-Rubin adjusted likelihood ratio test and lack clear interpretability. Furthermore, the 6-class and 7-class models have higher BIC scores than the 5-class solution, do not pass the Lo-Mendell-Rubin adjusted likelihood ratio tests, and lack parsimoniousness. | | | | | |

| **Supplementary Table 4**. Odds Ratios and Robust Standard Errors from Binary Logistic Regression and Ordered Logistic Regression Models Determining the Associations between Network Type and Subjective Well-being Indicators among Childless Individuals. | | | | | | | | | | | | | | | | | | | | | | | |
| --- | --- | --- | --- | --- | --- | --- | --- | --- | --- | --- | --- | --- | --- | --- | --- | --- | --- | --- | --- | --- | --- | --- | --- |
|  | | | | | | | | | | | | | | | | | | | | | | | |
|  | Lonely^a^ | | | | |  | Depressed^a^ | | | | |  | Happy^a^ | | | | |  | Life Satisfaction^b^ | | | | |
|  | Unadjusted model^c^ | |  | Adjusted model^c^ | |  | Unadjusted model^c^ | |  | Adjusted model^c^ | |  | Unadjusted model^c^ | |  | Adjusted model^c^ | |  | Unadjusted model^c^ | |  | Adjusted model^c^ | |
| Network type (Ref: Restricted) |  |  |  |  |  |  |  |  |  |  |  |  |  |  |  |  |  |  |  |  |  |  |  |
| Diverse, parental presence | 0.225 | *** |  | 0.335 | ** |  | 0.220 | *** |  | 0.198 | *** |  | 2.131 | ** |  | 1.545 |  |  | 3.946 | *** |  | 2.689 | *** |
|  | (0.093) |  |  | (0.155) |  |  | (0.094) |  |  | (0.098) |  |  | (0.636) |  |  | (0.559) |  |  | (1.229) |  |  | (1.008) |  |
| Diverse, parental absence | 0.792 |  |  | 1.480 |  |  | 0.345 | ** |  | 0.355 | ** |  | 4.015 | *** |  | 2.967 | *** |  | 5.058 | *** |  | 3.834 | *** |
|  | (0.273) |  |  | (0.664) |  |  | (0.145) |  |  | (0.176) |  |  | (1.380) |  |  | (1.196) |  |  | (1.697) |  |  | (1.459) |  |
| Parent-centered | 0.767 |  |  | 1.149 |  |  | 0.413 | ** |  | 0.447 | * |  | 1.246 |  |  | 1.018 |  |  | 3.042 | *** |  | 2.365 | ** |
|  | (0.249) |  |  | (0.460) |  |  | (0.154) |  |  | (0.194) |  |  | (0.375) |  |  | (0.361) |  |  | (1.036) |  |  | (0.885) |  |
| Siblings/extended family | 0.899 |  |  | 1.294 |  |  | 0.543 | ** |  | 0.590 |  |  | 1.674 | ** |  | 1.376 |  |  | 1.989 | *** |  | 2.010 | ** |
|  | (0.247) |  |  | (0.427) |  |  | (0.160) |  |  | (0.204) |  |  | (0.435) |  |  | (0.398) |  |  | (0.515) |  |  | (0.592) |  |
| **Sociodemographic characteristics** |  |  |  |  |  |  |  |  |  |  |  |  |  |  |  |  |  |  |  |  |  |  |  |
| Aged 65+ (Ref: 50-64) | 1.072 |  |  | 0.617 | * |  | 0.955 |  |  | 0.523 | ** |  | 1.056 |  |  | 1.646 | ** |  | 0.660 | ** |  | 1.158 |  |
|  | (0.223) |  |  | (0.168) |  |  | (0.229) |  |  | (0.158) |  |  | (0.196) |  |  | (0.388) |  |  | (0.119) |  |  | (0.255) |  |
| Female (Ref: Male) | 0.736 |  |  | 0.628 | * |  | 1.247 |  |  | 1.477 |  |  | 1.158 |  |  | 1.073 |  |  | 1.118 |  |  | 1.045 |  |
|  | (0.151) |  |  | (0.152) |  |  | (0.297) |  |  | (0.405) |  |  | (0.212) |  |  | (0.225) |  |  | (0.205) |  |  | (0.209) |  |
| Non-Chinese (Ref: Chinese) | 1.895 | ** |  | 1.804 | * |  | 1.480 |  |  | 1.298 |  |  | 1.216 |  |  | 1.658 |  |  | 1.355 |  |  | 1.341 |  |
|  | (0.478) |  |  | (0.636) |  |  | (0.425) |  |  | (0.514) |  |  | (0.294) |  |  | (0.546) |  |  | (0.358) |  |  | (0.453) |  |
| Foreign-born (Ref: Native-born) | 1.418 |  |  | 1.764 |  |  | 1.083 |  |  | 0.919 |  |  | 0.728 |  |  | 0.692 |  |  | 0.953 |  |  | 1.030 |  |
|  | (0.449) |  |  | (0.719) |  |  | (0.405) |  |  | (0.386) |  |  | (0.218) |  |  | (0.242) |  |  | (0.287) |  |  | (0.348) |  |
| Religion (Ref: Buddhism) |  |  |  |  |  |  |  |  |  |  |  |  |  |  |  |  |  |  |  |  |  |  |  |
| Non-Buddhism | 1.446 |  |  | 1.233 |  |  | 1.392 |  |  | 1.454 |  |  | 1.142 |  |  | 0.763 |  |  | 1.478 | * |  | 1.095 |  |
|  | (0.325) |  |  | (0.389) |  |  | (0.360) |  |  | (0.504) |  |  | (0.226) |  |  | (0.204) |  |  | (0.295) |  |  | (0.287) |  |
| No religion | 1.433 |  |  | 1.509 |  |  | 1.571 |  |  | 1.476 |  |  | 0.794 |  |  | 0.668 |  |  | 0.873 |  |  | 0.888 |  |
|  | (0.423) |  |  | (0.510) |  |  | (0.518) |  |  | (0.532) |  |  | (0.209) |  |  | (0.201) |  |  | (0.221) |  |  | (0.263) |  |
| Marital status (Ref: Currently married) |  |  |  |  |  |  |  |  |  |  |  |  |  |  |  |  |  |  |  |  |  |  |  |
| Never married | 2.217 | *** |  | 2.854 | *** |  | 1.459 |  |  | 1.789 |  |  | 0.884 |  |  | 0.592 | * |  | 0.551 | ** |  | 0.513 | ** |
|  | (0.645) |  |  | (1.076) |  |  | (0.455) |  |  | (0.778) |  |  | (0.198) |  |  | (0.178) |  |  | (0.131) |  |  | (0.157) |  |
| Widowed/divorced/  separated | 2.443 | ** |  | 2.002 |  |  | 1.812 |  |  | 1.296 |  |  | 0.729 |  |  | 0.783 |  |  | 0.331 | *** |  | 0.392 | *** |
|  | (0.951) |  |  | (0.912) |  |  | (0.764) |  |  | (0.630) |  |  | (0.242) |  |  | (0.295) |  |  | (0.100) |  |  | (0.140) |  |
| Involuntary childlessness | 1.137 |  |  | 1.397 |  |  | 1.540 |  |  | 1.753 |  |  | 0.651 | * |  | 0.548 | ** |  | 0.817 |  |  | 0.669 |  |
|  | (0.279) |  |  | (0.472) |  |  | (0.410) |  |  | (0.683) |  |  | (0.145) |  |  | (0.164) |  |  | (0.166) |  |  | (0.183) |  |
| Number of living siblings (Ref: Zero) |  |  |  |  |  |  |  |  |  |  |  |  |  |  |  |  |  |  |  |  |  |  |  |
| One-Two | 0.269 | *** |  | 0.227 | *** |  | 0.294 | *** |  | 0.432 | * |  | 1.804 | * |  | 1.372 |  |  | 1.471 |  |  | 0.658 |  |
|  | (0.099) |  |  | (0.100) |  |  | (0.115) |  |  | (0.203) |  |  | (0.638) |  |  | (0.562) |  |  | (0.554) |  |  | (0.292) |  |
| Three-Four | 0.499 | ** |  | 0.437 | ** |  | 0.359 | *** |  | 0.429 | * |  | 2.065 | ** |  | 1.673 |  |  | 1.336 |  |  | 0.723 |  |
|  | (0.171) |  |  | (0.177) |  |  | (0.133) |  |  | (0.186) |  |  | (0.716) |  |  | (0.649) |  |  | (0.487) |  |  | (0.307) |  |
| Five or more | 0.266 | *** |  | 0.187 | *** |  | 0.324 | *** |  | 0.388 | ** |  | 2.071 | ** |  | 1.636 |  |  | 1.011 |  |  | 0.524 |  |
|  | (0.095) |  |  | (0.080) |  |  | (0.122) |  |  | (0.176) |  |  | (0.719) |  |  | (0.642) |  |  | (0.366) |  |  | (0.221) |  |
| Education (Ref: Primary) |  |  |  |  |  |  |  |  |  |  |  |  |  |  |  |  |  |  |  |  |  |  |  |
| Secondary | 1.649 | * |  | 2.204 | ** |  | 1.207 |  |  | 1.239 |  |  | 1.506 | * |  | 1.350 |  |  | 2.045 | *** |  | 1.655 | * |
|  | (0.428) |  |  | (0.690) |  |  | (0.344) |  |  | (0.404) |  |  | (0.351) |  |  | (0.361) |  |  | (0.464) |  |  | (0.437) |  |
| Post-secondary | 0.912 |  |  | 1.587 |  |  | 0.685 |  |  | 0.718 |  |  | 1.845 | *** |  | 1.630 |  |  | 2.898 | *** |  | 1.965 | ** |
|  | (0.240) |  |  | (0.556) |  |  | (0.203) |  |  | (0.271) |  |  | (0.416) |  |  | (0.489) |  |  | (0.660) |  |  | (0.571) |  |
| Currently working (Ref: Not working) | 0.696 | * |  | 0.938 |  |  | 0.808 |  |  | 0.958 |  |  | 1.625 | *** |  | 1.526 | * |  | 1.924 | *** |  | 1.439 |  |
|  | (0.142) |  |  | (0.264) |  |  | (0.188) |  |  | (0.301) |  |  | (0.298) |  |  | (0.366) |  |  | (0.356) |  |  | (0.340) |  |
| Monthly income (Ref: Under $1,000) |  |  |  |  |  |  |  |  |  |  |  |  |  |  |  |  |  |  |  |  |  |  |  |
| $1,000-$1,999 | 0.703 |  |  | 0.564 | * |  | 0.747 |  |  | 0.676 |  |  | 1.509 | * |  | 1.242 |  |  | 1.336 |  |  | 1.002 |  |
|  | (0.183) |  |  | (0.178) |  |  | (0.225) |  |  | (0.235) |  |  | (0.366) |  |  | (0.346) |  |  | (0.327) |  |  | (0.260) |  |
| $2,000-$3,999 | 0.710 |  |  | 0.829 |  |  | 0.829 |  |  | 1.114 |  |  | 1.145 |  |  | 0.736 |  |  | 2.444 | *** |  | 1.376 |  |
|  | (0.197) |  |  | (0.302) |  |  | (0.260) |  |  | (0.413) |  |  | (0.293) |  |  | (0.231) |  |  | (0.672) |  |  | (0.440) |  |
| $4,000+ | 0.300 | *** |  | 0.357 | ** |  | 0.559 | * |  | 0.972 |  |  | 2.133 | *** |  | 1.329 |  |  | 3.413 | *** |  | 1.331 |  |
|  | (0.101) |  |  | (0.170) |  |  | (0.196) |  |  | (0.471) |  |  | (0.574) |  |  | (0.519) |  |  | (0.892) |  |  | (0.501) |  |
| Functional limitation (Ref: None) | 0.973 |  |  | 0.947 |  |  | 1.406 |  |  | 1.116 |  |  | 0.694 |  |  | 0.822 |  |  | 1.290 |  |  | 2.031 |  |
|  | (0.373) |  |  | (0.417) |  |  | (0.563) |  |  | (0.499) |  |  | (0.236) |  |  | (0.313) |  |  | (0.532) |  |  | (0.885) |  |
|  |  |  |  |  |  |  |  |  |  |  |  |  |  |  |  |  |  |  |  |  |  |  |  |
| Number |  | |  | 483 | |  |  | |  | 484 | |  |  | |  | 482 | |  |  | |  | 487 | |
| Note: * p<0.10, ** p<0.05, *** p<0.01; Robust standard errors in parentheses. | | | | | | | | | | | | | | | | | | | | | | | |
| ^a^ Subjective well-being indicators (lonely, depressed, happy) are assessed using binary logistic regressions. | | | | | | | | | | | | | | | | | | | | | | | |
| ^b^ Life satisfaction is assessed using ordered logistic regressions. | | | | | | | | | | | | | | | | | | | | | | | |
| ^c^ Unadjusted models include one covariate (i.e., zero-order effect). Adjusted models incorporate all covariates (network type and all sociodemographic characteristics). | | | | | | | | | | | | | | | | | | | | | | | |

| **Supplementary Table 5**. Odds Ratios and Robust Standard Errors from Binary Logistic Regression and Ordered Logistic Regression Models Determining the Associations between Network Type and Subjective Well-being Indicators among Childless and Non-Childless Individuals. | | | | | | | | | | | | | | | | | | | | | | | |
| --- | --- | --- | --- | --- | --- | --- | --- | --- | --- | --- | --- | --- | --- | --- | --- | --- | --- | --- | --- | --- | --- | --- | --- |
|  | | | | | | | | | | | | | | | | | | | | | | | |
|  | Lonely^a^ | | | | |  | Depressed^a^ | | | | |  | Happy^a^ | | | | |  | Life Satisfaction^b^ | | | | |
|  | Unadjusted model^c^ | |  | Adjusted model^c^ | |  | Unadjusted model^c^ | |  | Adjusted model^c^ | |  | Unadjusted model^c^ | |  | Adjusted model^c^ | |  | Unadjusted model^c^ | |  | Adjusted model^c^ | |
| Childlessness and network type (Ref: Non-childless) |  |  |  |  |  |  |  |  |  |  |  |  |  |  |  |  |  |  |  |  |  |  |  |
| Diverse, parental presence | 0.567 |  |  | 0.305 | *** |  | 0.610 |  |  | 0.393 | * |  | 1.210 |  |  | 1.688 |  |  | 0.919 |  |  | 1.054 |  |
|  | (0.205) |  |  | (0.132) |  |  | (0.233) |  |  | (0.204) |  |  | (0.274) |  |  | (0.539) |  |  | (0.208) |  |  | (0.328) |  |
| Diverse, parental absence | 1.996 | ** |  | 1.118 |  |  | 0.959 |  |  | 0.682 |  |  | 2.281 | *** |  | 3.071 | *** |  | 1.185 |  |  | 1.348 |  |
|  | (0.567) |  |  | (0.448) |  |  | (0.355) |  |  | (0.351) |  |  | (0.645) |  |  | (1.148) |  |  | (0.307) |  |  | (0.479) |  |
| Parent-centered | 1.933 | ** |  | 1.015 |  |  | 1.148 |  |  | 0.754 |  |  | 0.708 |  |  | 1.140 |  |  | 0.700 |  |  | 1.052 |  |
|  | (0.502) |  |  | (0.373) |  |  | (0.362) |  |  | (0.343) |  |  | (0.162) |  |  | (0.355) |  |  | (0.194) |  |  | (0.334) |  |
| Siblings/extended family | 2.267 | *** |  | 1.113 |  |  | 1.508 | * |  | 0.995 |  |  | 0.951 |  |  | 1.469 |  |  | 0.452 | *** |  | 0.809 |  |
|  | (0.435) |  |  | (0.372) |  |  | (0.332) |  |  | (0.405) |  |  | (0.163) |  |  | (0.414) |  |  | (0.073) |  |  | (0.223) |  |
| Restricted | 2.521 | *** |  | 1.167 |  |  | 2.776 | *** |  | 1.814 |  |  | 0.568 | *** |  | 0.990 |  |  | 0.222 | *** |  | 0.412 | *** |
|  | (0.579) |  |  | (0.400) |  |  | (0.652) |  |  | (0.762) |  |  | (0.122) |  |  | (0.306) |  |  | (0.052) |  |  | (0.142) |  |
| **Sociodemographic characteristics** |  |  |  |  |  |  |  |  |  |  |  |  |  |  |  |  |  |  |  |  |  |  |  |
| Aged 65+ (Ref: 50-64) | 0.911 |  |  | 0.706 | ** |  | 0.796 |  |  | 0.705 | ** |  | 0.977 |  |  | 1.326 | ** |  | 0.833 | * |  | 1.327 | ** |
|  | (0.119) |  |  | (0.111) |  |  | (0.116) |  |  | (0.121) |  |  | (0.102) |  |  | (0.169) |  |  | (0.088) |  |  | (0.172) |  |
| Female (Ref: Male) | 1.287 | * |  | 1.079 |  |  | 1.646 | *** |  | 1.727 | *** |  | 0.968 |  |  | 1.048 |  |  | 0.929 |  |  | 1.133 |  |
|  | (0.172) |  |  | (0.164) |  |  | (0.251) |  |  | (0.290) |  |  | (0.102) |  |  | (0.123) |  |  | (0.101) |  |  | (0.135) |  |
| Non-Chinese (Ref: Chinese) | 1.740 | *** |  | 1.890 | *** |  | 1.651 | *** |  | 1.799 | ** |  | 1.591 | *** |  | 1.578 | ** |  | 1.645 | *** |  | 1.493 | ** |
|  | (0.254) |  |  | (0.410) |  |  | (0.265) |  |  | (0.419) |  |  | (0.206) |  |  | (0.280) |  |  | (0.213) |  |  | (0.260) |  |
| Foreign-born (Ref: Native-born) | 0.870 |  |  | 1.005 |  |  | 0.807 |  |  | 0.785 |  |  | 1.131 |  |  | 1.082 |  |  | 0.928 |  |  | 0.749 | ** |
|  | (0.152) |  |  | (0.195) |  |  | (0.159) |  |  | (0.164) |  |  | (0.155) |  |  | (0.159) |  |  | (0.125) |  |  | (0.108) |  |
| Religion (Ref: Buddhism) |  |  |  |  |  |  |  |  |  |  |  |  |  |  |  |  |  |  |  |  |  |  |  |
| Non-Buddhism | 1.394 | ** |  | 0.920 |  |  | 1.416 | ** |  | 1.061 |  |  | 1.540 | *** |  | 1.201 |  |  | 1.763 | *** |  | 1.349 | ** |
|  | (0.196) |  |  | (0.186) |  |  | (0.223) |  |  | (0.227) |  |  | (0.174) |  |  | (0.181) |  |  | (0.203) |  |  | (0.205) |  |
| No religion | 1.117 |  |  | 1.067 |  |  | 1.447 | * |  | 1.543 | * |  | 1.258 |  |  | 1.232 |  |  | 1.218 |  |  | 1.171 |  |
|  | (0.225) |  |  | (0.226) |  |  | (0.309) |  |  | (0.342) |  |  | (0.198) |  |  | (0.207) |  |  | (0.205) |  |  | (0.206) |  |
| Marital status (Ref: Currently married) |  |  |  |  |  |  |  |  |  |  |  |  |  |  |  |  |  |  |  |  |  |  |  |
| Never married | 2.81 | *** |  | 2.747 | *** |  | 1.545 | ** |  | 1.500 |  |  | 0.887 |  |  | 0.671 |  |  | 0.445 | *** |  | 0.598 | * |
|  | (0.444) |  |  | (0.831) |  |  | (0.266) |  |  | (0.563) |  |  | (0.115) |  |  | (0.174) |  |  | (0.063) |  |  | (0.157) |  |
| Widowed/divorced/  separated | 2.576 | *** |  | 2.138 | *** |  | 1.372 | * |  | 1.016 |  |  | 0.739 | ** |  | 0.798 |  |  | 0.503 | *** |  | 0.667 | *** |
|  | (0.415) |  |  | (0.398) |  |  | (0.245) |  |  | (0.216) |  |  | (0.097) |  |  | (0.121) |  |  | (0.067) |  |  | (0.102) |  |
| Involuntary childlessness | 1.696 | ** |  | 1.617 |  |  | 1.820 | ** |  | 1.839 |  |  | 0.669 | * |  | 0.516 | ** |  | 0.545 | *** |  | 0.641 |  |
|  | (0.385) |  |  | (0.514) |  |  | (0.440) |  |  | (0.720) |  |  | (0.137) |  |  | (0.149) |  |  | (0.106) |  |  | (0.177) |  |
| Number of living siblings (Ref: Zero) |  |  |  |  |  |  |  |  |  |  |  |  |  |  |  |  |  |  |  |  |  |  |  |
| One-Two | 0.716 |  |  | 1.011 |  |  | 0.657 |  |  | 0.923 |  |  | 1.447 | * |  | 1.034 |  |  | 1.362 |  |  | 0.815 |  |
|  | (0.177) |  |  | (0.275) |  |  | (0.175) |  |  | (0.277) |  |  | (0.313) |  |  | (0.246) |  |  | (0.296) |  |  | (0.188) |  |
| Three-Four | 0.755 |  |  | 0.969 |  |  | 0.634 | * |  | 0.752 |  |  | 1.569 | ** |  | 1.191 |  |  | 1.234 |  |  | 0.822 |  |
|  | (0.180) |  |  | (0.247) |  |  | (0.163) |  |  | (0.215) |  |  | (0.330) |  |  | (0.272) |  |  | (0.250) |  |  | (0.178) |  |
| Five or more | 0.534 | *** |  | 0.700 |  |  | 0.532 | ** |  | 0.641 |  |  | 1.680 | ** |  | 1.305 |  |  | 1.168 |  |  | 0.780 |  |
|  | (0.128) |  |  | (0.180) |  |  | (0.136) |  |  | (0.183) |  |  | (0.347) |  |  | (0.293) |  |  | (0.231) |  |  | (0.164) |  |
| Education (Ref: Primary) |  |  |  |  |  |  |  |  |  |  |  |  |  |  |  |  |  |  |  |  |  |  |  |
| Secondary | 0.946 |  |  | 1.029 |  |  | 0.881 |  |  | 0.848 |  |  | 1.579 | *** |  | 1.416 | ** |  | 1.883 | *** |  | 1.471 | *** |
|  | (0.147) |  |  | (0.177) |  |  | (0.150) |  |  | (0.157) |  |  | (0.203) |  |  | (0.199) |  |  | (0.245) |  |  | (0.210) |  |
| Post-secondary | 0.690 | ** |  | 1.032 |  |  | 0.664 | ** |  | 0.708 |  |  | 1.708 | *** |  | 1.365 | * |  | 2.765 | *** |  | 1.914 | *** |
|  | (0.112) |  |  | (0.213) |  |  | (0.119) |  |  | (0.149) |  |  | (0.220) |  |  | (0.226) |  |  | (0.380) |  |  | (0.327) |  |
| Currently working (Ref: Not working) | 0.784 | * |  | 0.891 |  |  | 0.955 |  |  | 1.054 |  |  | 1.312 | *** |  | 1.180 |  |  | 1.348 | *** |  | 0.982 |  |
|  | (0.102) |  |  | (0.144) |  |  | (0.137) |  |  | (0.188) |  |  | (0.137) |  |  | (0.155) |  |  | (0.143) |  |  | (0.131) |  |
| Monthly income (Ref: Under $1,000) |  |  |  |  |  |  |  |  |  |  |  |  |  |  |  |  |  |  |  |  |  |  |  |
| $1,000-$1,999 | 0.75 | * |  | 0.850 |  |  | 0.874 |  |  | 0.968 |  |  | 1.260 |  |  | 1.094 |  |  | 1.511 | *** |  | 1.342 | * |
|  | (0.128) |  |  | (0.163) |  |  | (0.171) |  |  | (0.211) |  |  | (0.190) |  |  | (0.179) |  |  | (0.236) |  |  | (0.215) |  |
| $2,000-$3,999 | 0.541 | *** |  | 0.746 |  |  | 0.880 |  |  | 1.134 |  |  | 1.227 |  |  | 0.992 |  |  | 2.269 | *** |  | 1.764 | *** |
|  | (0.098) |  |  | (0.169) |  |  | (0.174) |  |  | (0.274) |  |  | (0.186) |  |  | (0.182) |  |  | (0.360) |  |  | (0.318) |  |
| $4,000+ | 0.299 | *** |  | 0.443 | *** |  | 0.526 | *** |  | 0.746 |  |  | 1.807 | *** |  | 1.392 |  |  | 3.731 | *** |  | 2.422 | *** |
|  | (0.062) |  |  | (0.124) |  |  | (0.115) |  |  | (0.220) |  |  | (0.278) |  |  | (0.303) |  |  | (0.614) |  |  | (0.529) |  |
| Functional limitation (Ref: None) | 0.909 |  |  | 0.818 |  |  | 1.689 | ** |  | 1.634 | * |  | 0.946 |  |  | 1.042 |  |  | 1.055 |  |  | 1.254 |  |
|  | (0.215) |  |  | (0.207) |  |  | (0.379) |  |  | (0.420) |  |  | (0.175) |  |  | (0.205) |  |  | (0.216) |  |  | (0.270) |  |
|  |  |  |  |  |  |  |  |  |  |  |  |  |  |  |  |  |  |  |  |  |  |  |  |
| Number |  | |  | 483 | |  |  | |  | 484 | |  |  | |  | 482 | |  |  | |  | 487 | |
| Note: * p<0.10, ** p<0.05, *** p<0.01; Robust standard errors in parentheses. | | | | | | | | | | | | | | | | | | | | | | | |
| ^a^ Subjective well-being indicators (lonely, depressed, happy) are assessed using binary logistic regressions. | | | | | | | | | | | | | | | | | | | | | | | |
| ^b^ Life satisfaction is assessed using ordered logistic regressions. | | | | | | | | | | | | | | | | | | | | | | | |
| ^c^ Unadjusted models include one covariate (i.e., zero-order effect). Adjusted models incorporate all covariates (network type and all sociodemographic characteristics). | | | | | | | | | | | | | | | | | | | | | | | |
